# Interaction with Mesenchymal Stem Cells Provokes Natural Killer Cells for Enhanced IL-12/IL-18-Induced Interferon-Gamma Secretion

**DOI:** 10.1155/2014/143463

**Published:** 2014-04-30

**Authors:** Heike Thomas, Marcus Jäger, Katharina Mauel, Sven Brandau, Sara Lask, Stefanie B. Flohé

**Affiliations:** ^1^Orthopedic Research, Department of Orthopedics, University Hospital Essen, University Duisburg-Essen, Virchowstraße 171, 45147 Essen, Germany; ^2^Surgical Research, Department of Trauma Surgery, University Hospital Essen, University Duisburg-Essen, Virchowstraße 171, 45147 Essen, Germany; ^3^Department of Otolaryngology, University Hospital Essen, University Duisburg-Essen, Virchowstraße 171, 45147 Essen, Germany

## Abstract

Tissue injury induces an inflammatory response accompanied by the recruitment of immune cells and of mesenchymal stem cells (MSC) that contribute to tissue regeneration. After stimulation with interleukin- (IL-) 12 and IL-18 natural killer (NK) cells secrete the proinflammatory cytokine interferon- (IFN-) **γ**. IFN-**γ** plays a crucial role in the defense against infections and modulates tissue regeneration. In consideration of close proximity of NK cells and MSC at the site of injury we investigated if MSC could influence the ability of NK-cells to produce IFN-**γ**. Coculture experiments were performed with bone marrow-derived human MSC and human NK cells. MSC enhanced the ability of IL-12/IL-18-stimulated NK cells to secrete IFN-**γ** in a dose-dependent manner. This activation of NK cells was dependent on cell-cell contact as well as on soluble factors. The increased IFN-**γ** secretion from NK cells after contact with MSC correlated with an increased level of intracellular IFN-**γ**. Alterations in the IL-12 signaling pathway including an increased expression of the IL-12*β*1 receptor subunit and an increased phosphorylation of signal transducer and activator of transcription 4 (STAT4) could be observed. In conclusion, MSC enhance the IFN-**γ** release from NK cells which might improve the defense against infections at the site of injury but additionally might affect tissue regeneration.

## 1. Introduction

Mesenchymal stem cells (MSC) are multipotent adult stem cells which are present in a variety of tissues. The most important source of MSC is the bone marrow. The cells grow rapidly in culture and due to their multilineage differentiation capacity they have a fundamental role in the repair and regeneration of injured mesenchymal tissue, like bone, cartilage, muscle, adipose tissue, and connective tissue. Therefore, MSC have a therapeutic potential and are clinically used to treat bone and cartilage damages, cardiovascular defects, and ligamentous injuries (summarized in [1]).

Natural killer (NK) cells are part of the innate immune system, evolve as progenitors in the bone marrow, and circulate as mature cells in the blood. They play a key role in the elimination of virus-infected cells as well as in controlling tumor cell growth. Their function is mainly regulated by activating or inhibiting cell surface receptors transmitting the signal into the cell [2]. In addition, NK cells possess regulatory functions and can secrete cytokines and chemokines which modulate the host's immune response. One relevant cytokine expressed by NK cells is interferon- (IFN-) *γ*, which is produced upon stimulation with interleukin- (IL-) 12 that is released from accessory cells like monocytes, macrophages, and dendritic cells (DC). Other cytokines like IL-1, IL-2, IL-15, or IL-18 potentiate the activity of IL-12 [3–5]. IL-12 is one of the most important proinflammatory cytokines that is rapidly released in response to penetrating pathogens and acts through its high affinity receptor. The IL-12 receptor (IL-12R) is a heterodimer composed of the *β*1 and *β*2 subunits, which are both required to generate a functional receptor [6]. Both IL-12 and IL-18 cause a reciprocal upregulation of their receptors and synergistically activate the expression of the IFN-*γ* gene through different signaling pathways [7, 8]. The transcription of the IFN-*γ* gene requires the activation of transcription factors like nuclear factor kappa-light-chain-enhancer of activated B-cells (NF*κ*B), signal transducer and activator of transcription 4 (STAT4), and activator protein- (AP-) 1 [7, 9]. Among these factors, STAT4 plays a central role in IL-12 signaling. STAT4 knockout mice lack all major IL-12-induced functions in T-cells as well as in NK cells, including the ability to produce IFN-*γ* [10]. The NK cell-derived IFN-*γ* reinforces via a feedback mechanism the expression of IL-12 from DC [11, 12]. The release of IFN-*γ* can occur within minutes. In addition to its key role in the defense against infections, IFN-*γ* also plays a functional role in the process of tissue regeneration, as it is required for instance for skeletal muscle [13] and bone regeneration [14, 15].

Due to their capacity to lyse target cells, to secrete immunomodulatory cytokines, and to interact with other cells, NK cells possess multiple functions. In addition to the defense against pathogens, NK cells also play an important role in the repair and regeneration of damaged tissue [16]. NK cells are rapidly recruited to the site of injury where they might come in contact with MSC. Interactions between MSC and NK cells might exert relevant effects on the function of both cell types. Understanding the mechanism of the interaction is of great importance for therapeutic approaches.

In addition to their role in repair and regeneration, MSC possess immunomodulatory properties. Therefore, they are successfully used to treat immune-related disorders including graft versus host disease in patients after hematopoietic stem cell transplantation [17], Crohn's disease, or multiple sclerosis [18]. It is well described that MSC suppress the proliferation and function of cells of the adaptive immune system like T-lymphocytes, *γδ*T-cells, NKT-cells, and B-cells (summarized in [19]). Several studies investigated the influence of MSC on the proliferation and the cytolytic activity of NK cells and described inhibitory effects [20–22]. But conflicting data exist on the influence of MSC on the IFN-*γ* production of NK cells; some studies [22–24] reported an increased IFN-*γ* production by NK cells and other studies [20, 21, 25] described a reduced IFN-*γ* production by NK cells due to the presence of MSC.

As IL-12 is a very important proinflammatory cytokine rapidly released by immune cells, for example, during infection, and the effect of MSC on NK cells in the IL-12-containing cytokine milieu has not been examined so far, we investigated the influence of bone marrow-derived MSC on the IL-12/IL-18-stimulated IFN-*γ* production of NK cells. In the present report, we show that MSC enhanced the IL-12/IL-18-induced IFN-*γ* production from NK cells. This modulatory activity of MSC was mediated via cell-cell contact as well as by soluble factors and was associated with an activation of the IL-12R/STAT4 signaling pathway in NK cells.

## 2. Materials and Methods

### 2.1. Culture of Human MSC

Following approval of the local ethics committee and informed consent, human MSC were obtained from the bone marrow aspirates of patients who underwent total hip replacement surgery. The bone marrow aspirate was dissociated with Dulbecco phosphate-buffered saline (DPBS) and centrifuged at 300 g for 10 minutes at room temperature. Cells were resuspended in DPBS and mononuclear cells were isolated by Ficoll-Paque Plus (GE Healthcare Life Sciences, Freiburg, Germany) density gradient centrifugation. Subsequently, cells were cultured in MSC medium composed of Dulbecco's modified Eagle's medium (DMEM; Gibco life technologies, Darmstadt, Germany) supplemented with 10% fetal calf serum (FCS, Biochrom, Berlin, Germany), 100 U/mL penicillin, 0.1 mg/mL streptomycin, 2 mM L-glutamine, and 1 mM sodium pyruvate and were incubated in a humidified atmosphere containing 5% CO_2_ at 37°C. After 24 h, nonadherent cells were removed and fresh medium was added. Half the medium was replaced twice a week. When the cultures nearly reached confluence, cells were detached by treatment with Accutase (Gibco) and were replated in 1 : 2 dilutions. MSC were used in the experiments in passages 3–9.

Adherent cells in passage 3 were characterized by flow cytometric analysis for the expression of the typical surface markers CD73, CD90, and CD105 and the absence of the hematopoietic markers CD34 and CD45. The ability of MSC to differentiate into adipocytes, chondrocytes, and osteocytes was confirmed by immunohistochemistry (data not shown).

### 2.2. Culture of NK Cells

The human NK92 cell line was purchased from DSMZ (Braunschweig, Germany). The cells were cultured in medium VLE RPMI 1640 (Biochrom) supplemented with 12.5% fetal calf serum (FCS Gold, PAA Laboratories, Pasching, Austria), 12.5% horse serum (HS, PAN Biotech, Aidenbach, Germany), 2 mM L-glutamine, 100 U/mL penicillin, 0.1 mg/mL streptomycin, and 100 U/mL IL-2 (PeproTech, Hamburg, Germany) at 37°C in a humidified atmosphere containing 5% CO_2_. Cell cultures were split 1 : 2 every 2 days.

Primary human NK cells were isolated from peripheral blood of healthy volunteers. Peripheral blood mononuclear cells (PBMCs) were obtained by Ficoll-Paque Plus (GE Healthcare Life Sciences) density gradient centrifugation. Subsequently, NK cells were isolated from PBMC using the human PAN NK cell isolation kit (MACS, Miltenyi Biotec, Bergisch-Gladbach, Germany) according to the manufacturer's instructions. The isolated cell fraction contained negligible amounts of CD3^+^ T-lymphocytes and >90% of CD56^+^ NK cells as determined by flow cytometry. Purified NK cells were used directly for coculture experiments.

### 2.3. MSC-NK Cell Coculture

Human MSC (10^4^, 2 × 10^3^, 10^3^, or 5 × 10^2^ cells per well) were plated in triplicate in 96-well plates in MSC medium and were allowed to adhere to the plate for 24 h. Medium was changed and NK92 cells or primary NK cells, as indicated, (10^4^ cells per well) were added in RPMI 1640 medium lacking IL-2. The NK cell to MSC ratio varied from 20 : 1 to 1 : 1. Subsequently or 24 h later IL-12 (1 ng/mL) and IL-18 (10 ng/mL) were added for further 24, 48, or 72 h as indicated in the figure legends.

Transwell experiments were performed in 24-well transwell plates (0.4 *μ*m pore size, BD Biosciences). MSC (1.25 × 10^4^ cells per well) were seeded in the lower chamber. Twenty-four hours later 6.25 × 10^4^ NK cells per well were added in the upper chamber and after 24 h IL-12 (1 ng/mL) and IL-18 (10 ng/mL) were added for further 24 h. To ensure the correct concentration in both chambers, IL-12 and IL-18 were added proportionally to both chambers.

To obtain conditioned media, MSC (1.25 × 10^4^ cells per well) were seeded in 24-well plates using MSC medium. 24 h later NK92 cells (6.25 × 10^4^ cells per well) were added in RPMI 1640 medium lacking IL-2. Yet another 24 h later, the supernatant of the cocultures was taken off and residual cells were removed by centrifugation. Finally, this supernatant was used as conditioned medium that was added to fresh NK-92 cells (10^4^ cells per well) in a 96-well plate before stimulation with IL-12 and IL-18 as described above.

The supernatant of the cell cultures was harvested, depleted from residual cells by centrifugation, and stored at −20°C until use for enzyme-linked immunosorbent assay (ELISA).

### 2.4. Quantification of IFN-*γ* by ELISA

Quantification of human IFN-*γ* in cell culture supernatants was performed using a commercially available ELISA kit (Duo Set, R&D Systems, Wiesbaden, Germany) according to the manufacturer's instructions. The detection range was given at 15.6–1000 pg/mL.

### 2.5. Monoclonal Antibodies and Flow Cytometry

The following monoclonal antibodies (mAbs) were used in this study: CD56-Allophycocyanin (APC) (Clone CMSSB; eBioscience, Frankfurt, Germany), IFN-*γ*-Phycoerythrin (PE) (Clone 4S.B3; IgG1, *κ*; BioLegend, San Diego, CA, USA); STAT4-PE (Clone 38/p-STAT4; IgG2b, *κ*; BD Biosciences, Heidelberg, Germany), and CD212-PE (Clone 2.4E6; IgG1, *κ*; BD Biosciences). To analyze the surface IL-12 receptor (IL-12R/CD212) of NK cells, cell samples were stained with the appropriate mAbs as described [26]. To analyze intracellular pSTAT4, NK cells were first stained with mAbs against surface CD56 and then fixed and permeabilized using the fixation/permeabilization buffer set (eBioscience) according to the manufacturer's instructions. Thereafter, mAbs against pSTAT4 were added and incubated for 30 minutes at 4°C. For intracellular detection of IFN-*γ*, 0.66 *μ*L/mL monensin (GolgiStop, BD Biosciences) was added during the last 6 h of coculture. After surface staining of CD56, cells were fixed and permeabilized using Cytofix/Cytoperm (BD Biosciences) before addition of mAbs against IFN-*γ*. Appropriate isotype controls of pooled NK-cell cultures in the absence or in the presence of MSC were used for all stainings. Flow cytometry was performed using a FACS Calibur (BD Bioscience). Data were analyzed using Cell Quest Pro software (BD Bioscience).

### 2.6. Statistical Analysis

All data were expressed as mean value ± standard deviation and statistical analyses were performed using Microsoft Excel2010 or GraphPad Prism5.0. Statistical differences were tested using the one-way ANOVA followed by Bonferroni multiple comparison test or a paired *t*-test. A *P* value < 0.05 was considered significant.

## 3. Results 

### 3.1. MSC Potentiate the IL-12/IL-18-Induced IFN-*γ* Secretion by NK Cells

**Figure 1:**
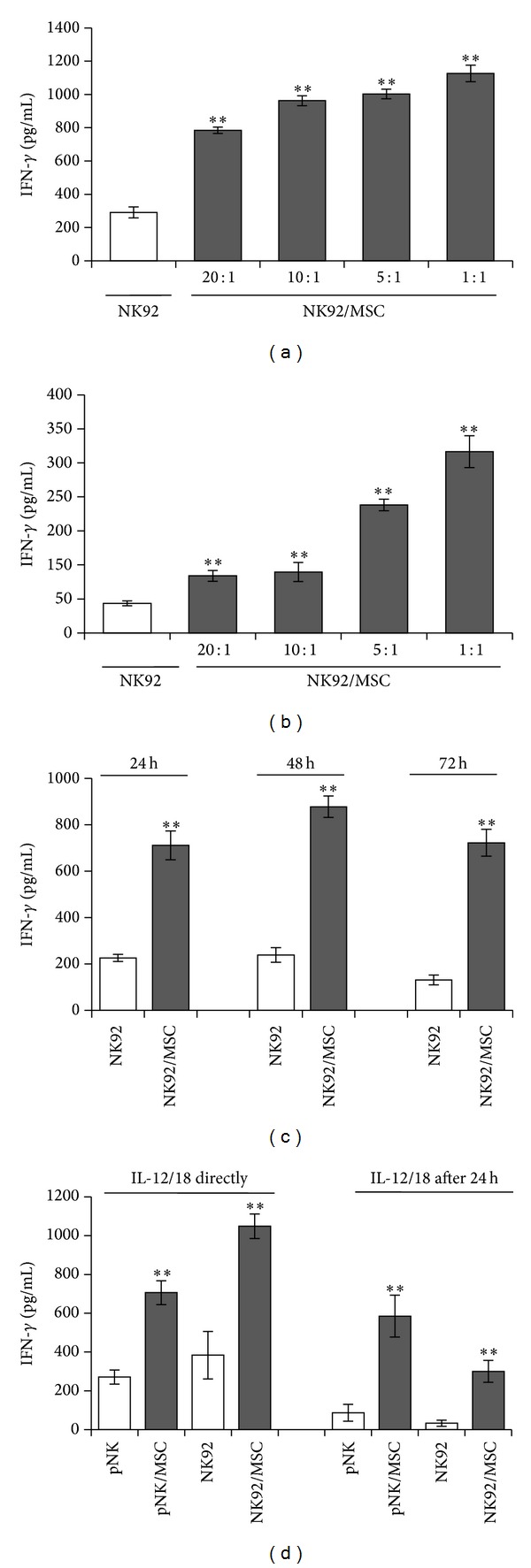
MSC exert a dose-dependent activating effect on IFN-*γ* secretion by NK92 cells. NK92 cells were cultured in the absence or presence of MSC at NK : MSC ratios of 20 : 1, 10 : 1, 5 : 1, and 1 : 1 (a and b) or 5 : 1 (c and d). Interleukin- (IL-) 12 (1 ng/mL) and IL-18 (10 ng/mL) were added directly after seeding the NK92 cells (a and c) or 24 h after preincubation of MSC and NK cells (b). After 24 h, the IFN-*γ* concentration in the supernatants was quantified by ELISA. Supernatants derived from unstimulated NK-cell cultures in the absence or in the presence of MSC show no detectable IFN-*γ*. Bar graphs show mean values ± standard deviation of triplicate cultures from one representative experiment out of three independent experiments done with three different donors for MSC. **P < 0.01 versus NK cells cultured alone.

As the release of immunoregulatory cytokines like IFN-*γ* plays a key role in NK cell function, the influence of MSC on IFN-*γ* secretion by NK cells was investigated. Therefore, coculture experiments of varying numbers of primary human MSC and constant numbers of the human cell line NK92 were performed to determine the IL-12/IL-18-induced IFN-*γ* secretion by NK cells. In the presence of increasing numbers of MSC, NK92 cells secreted 2.5-fold to 4-fold more IFN-*γ* than NK cells alone (Figure 1(a) and Table 1). This stimulating effect of MSC was even more pronounced when the NK cells were incubated with MSC for 24 h prior to stimulation with IL-12/IL-18 (Figure 1(b) and Table 1). The ratio of 5 : 1 (NK : MSC) was selected for further experiments. To clarify whether the MSC-mediated increase in the IFN-*γ* secretion of NK92 cells was due to altered kinetics of IFN-*γ* production the coculture experiments were prolonged over a period of 72 h. At any time point the presence of MSC significantly increased the IFN-*γ* secretion from NK cells (Figure 1(c) and Table 1). To exclude that the effect of MSC was specific for the NK cell line only, similar experiments were performed with primary NK cells isolated from human peripheral blood. Similar to NK92 cells primary NK cells released increased levels of IFN-*γ* when stimulated in the presence of MSC (Figure 1(d) and Table 1). Remarkably, no IFN-*γ* was detectable in supernatants derived from NK-cell-cultures in the absence of IL-12/IL-18. In summary, MSC potentiate the IL-12/IL-18-induced IFN-*γ* secretion by NK cells in a dose-dependent manner and at any time point within a period of three days.

### 3.2. MSC Prime the IFN-*γ* Secretion by NK92 Cells through Soluble Factors and Cell-Cell Contact

**Figure 2:**
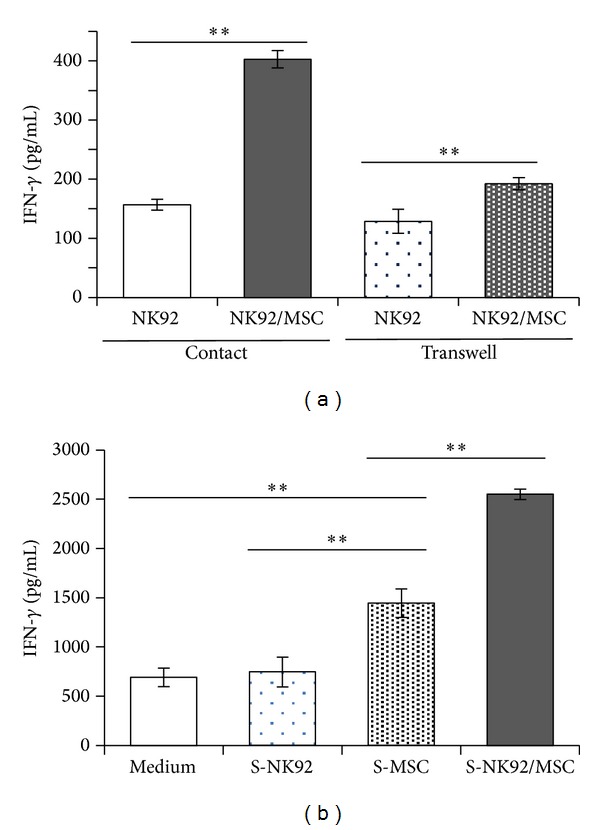
MSC promote the secretion of IFN-*γ* by NK92 cells both by cell-cell contact as well as by soluble factors. (a) NK92 cells were cultured in the absence or presence of MSC at a NK : MSC ratio of 5 : 1 in a contact or a transwell system. IL-12 (1 ng/mL) and IL-18 (10 ng/mL) were added 24 h after preincubation of MSC and NK cells. (b) NK92 cells were cultured in the absence or presence of MSC at a NK : MSC ratio of 5 : 1. After 24 h the supernatant (S) was harvested and transferred to fresh NK92 cells prior to stimulation with IL-12 (1 ng/mL) and IL-18 (10 ng/mL) for 24 h. The IFN-*γ* concentration in the supernatants was quantified by ELISA. Bar graphs show mean values ± standard deviation of triplicate cultures from one representative experiment out of three independent experiments done with three different donors for MSC. **P < 0.01.

In order to investigate whether cell-cell contact was required for MSC-mediated NK cell activation cocultures were performed in a transwell system where both cell types are separated by a membrane permeable to soluble molecules. As shown in Figure 2(a) MSC provoked a 2.5-fold increase in IFN-*γ* secretion by NK92 cells when MSC and NK cells were in close contact compared with NK cells alone. However, upon spatial separation of both cell types MSC still caused an increased IFN-*γ* secretion from NK92 cells but to a lesser extent (Figure 2(a)). To address the potential role of soluble factors in MSC-mediated NK cell activation, conditioned media from MSC alone, NK cells alone, and NK/MSC cocultures in the absence of any stimulus were prepared and added to fresh NK92 cell cultures before stimulation with IL-12 and IL-18. As illustrated in Figure 2(b) the IFN-*γ* secretion from NK92 cells was not affected by the presence of conditioned medium prepared from NK92 cells alone. However, in the presence of conditioned medium prepared from MSC alone, the IFN-*γ* secretion from NK92 cells was significantly increased (Figure 2(b)). The conditioned medium from NK/MSC cocultures was even more potent in NK cell stimulation (Figure 2(b)). In conclusion, the stimulation of NK cells by MSC is mediated by both cell-cell contact and soluble factors derived from MSC.

### 3.3. MSC Increase Intracellular IFN-*γ* Production of NK Cells

**Figure 3:**
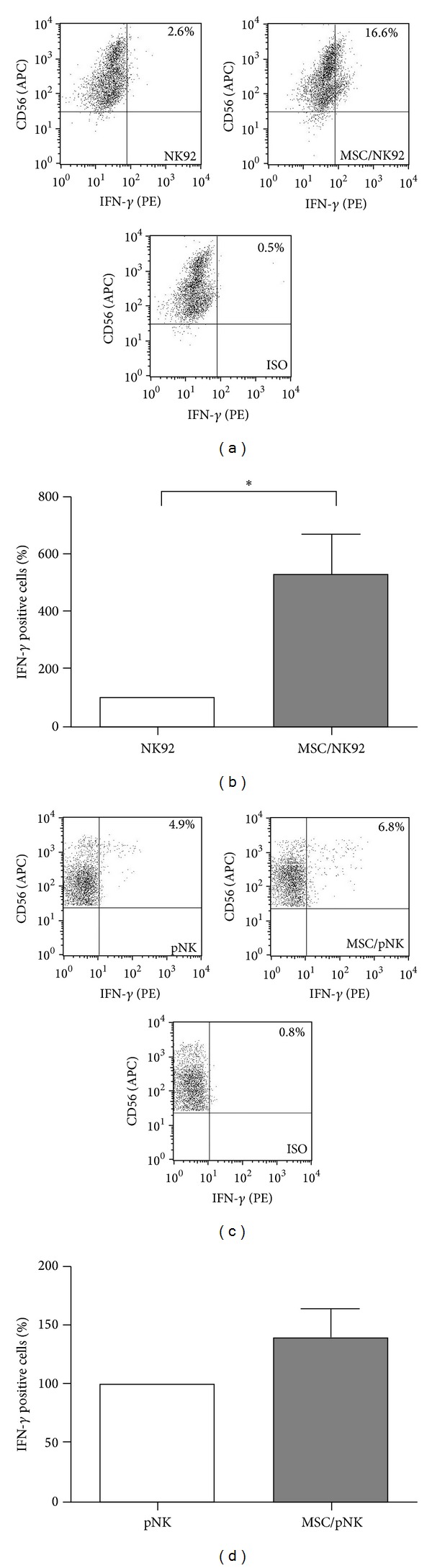
MSC promote the intracellular IFN-*γ* production in NK cells. NK92 cells ((a) and (b)) and primary NK- (pNK) cells ((c) and (d)) were cultured in the absence or presence of MSC at a NK : MSC ratio of 5 : 1. Cells were preincubated for 24 h prior to stimulation with IL-12 (1 ng/mL) and IL-18 (10 ng/mL). Sixteen hours after stimulation, the cells were incubated with GolgiStop for 6 h and subsequently stained with antibodies against CD56 and intracellular IFN-*γ* before flow cytometric analysis. ((a) and (c)) Dot plots of one representative experiment. ((b) and (d)) Bar graphs show mean values ± standard deviation of 6 (b) and 5 (d) independent experiments performed using (b) 6 different donors for MSC and (d) 3 and 4 different donors for MSC and pNK cells, respectively. Data are normalized and presented as percentage of IFN-*γ* positive NK cells cultured in the presence of MSC with respect to NK cells cultured alone (100%). *P < 0.05.

To test whether the rising IFN-*γ* secretion of NK cells upon coculture with MSC resulted from an increased IFN-*γ* production per cell, intracellular staining of IFN-*γ* in NK cells was performed after coculture of MSC and NK cells and stimulation with IL-12 and IL-18. As shown in Figure 3(a), 2.6% of the whole NK92 cell population produced IFN-*γ* in the absence of MSC. After coculture with MSC, IFN-*γ* synthesis was detected in almost 17% of NK92 cells (Figure 3(a)). The extent of MSC-mediated stimulation of NK92 cells varied depending on the donor of MSC but significantly increased more than 5-fold (Figure 3(b)). Using primary NK cells 4.9% of the cells produced IFN-*γ* in the absence of MSC. After coculture with MSC, there was a slight increase to 6.8% (Figure 3(c)). The extent of MSC-mediated stimulation of primary NK cells was lower compared to NK92 cells and was around 1.5-fold (Figure 3(d)). Thus, MSC stimulate NK cells for increased intracellular production and secretion of IFN-*γ*.

### 3.4. MSC Trigger the IL-12/STAT4 Signaling Pathway in NK Cells

**Figure 4:**
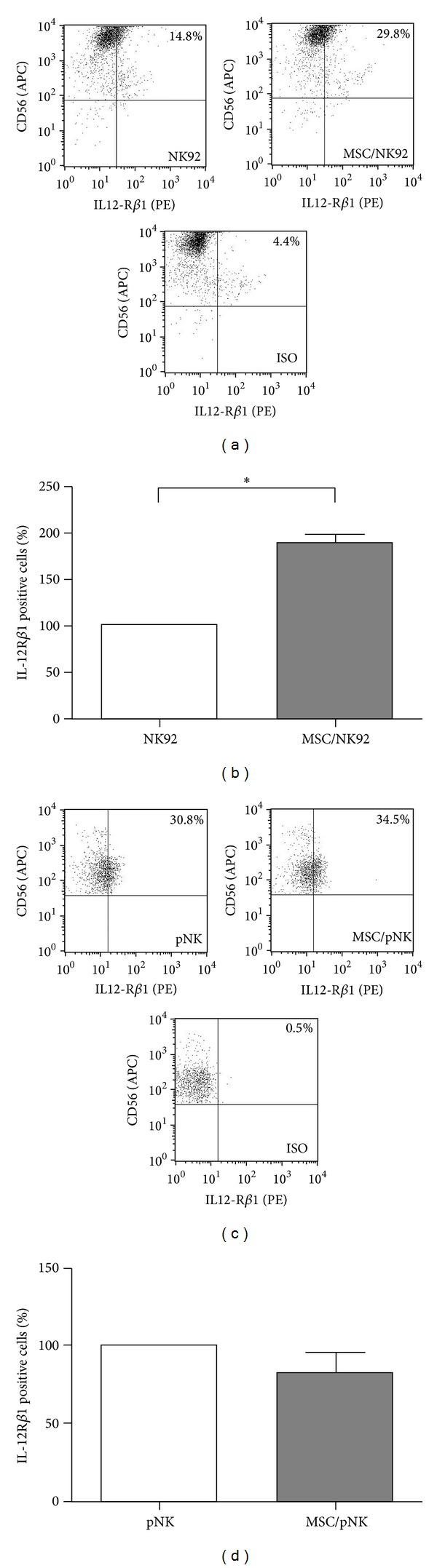
MSC stimulate the expression of IL-12R*β*1 on NK cells. NK92 cells ((a) and (b)) and primary NK- (pNK) cells ((c) and (d)) were cultured in the absence or presence of MSC for 24 h at a NK : MSC ratio of 5 : 1. Cells were stained with antibodies against CD56 and IL-12R*β*1. Surface IL-12R expression was measured by flow cytometry. ((a) and (c)) Dot plots of one representative experiment. ((b) and (d)) Bar graph shows mean values ± standard deviation of 6 independent experiments performed using (b) 6 different donors for MSC and (d) 3 and 4 different donors for MSC and pNK cells, respectively. Data are normalized and presented as percentage of IL-12R*β*1 positive NK cells cultured in the presence of MSC with respect to NK cells cultured alone (100%). *P < 0.05.

To address potential mechanisms underlying the MSC-mediated effect on NK cells, we investigated the IL-12/STAT4 signaling pathway in NK cells in the presence or absence of MSC. We explored the expression of the cell surface IL-12R and of intracellular phosphorylated STAT4 (pSTAT4) proteins. Therefore, NK cells were cocultured in the presence or absence of MSC for 24 h and the expression of the IL-12R*β*1 chain was determined by flow cytometry. The expression of the IL-12R*β*1 chain increased from 14.8% to 29.8% on the surface on NK92 cells alone and in the presence of MSC, respectively (Figure 4(a)). This reflects a rising expression of this receptor chain in the whole NK92 cell population rather than an increase per cell. As shown in Figure 4(b) the results of a total of 6 experiments confirmed this rising IL-12R*β*1 expression in the presence of MSC from different donors showing an average stimulation of nearly 2-fold. Using primary NK cells, a slight increase in the expression of IL-12R*β*1 was observed after coculture with MSC (Figure 4(c)) depending on the NK cell donor. Nevertheless, considering the entire set of experiments expression of the *β*1 chain was not significantly increased in the absence and presence of MSC (Figure 4(d)).

**Figure 5:**
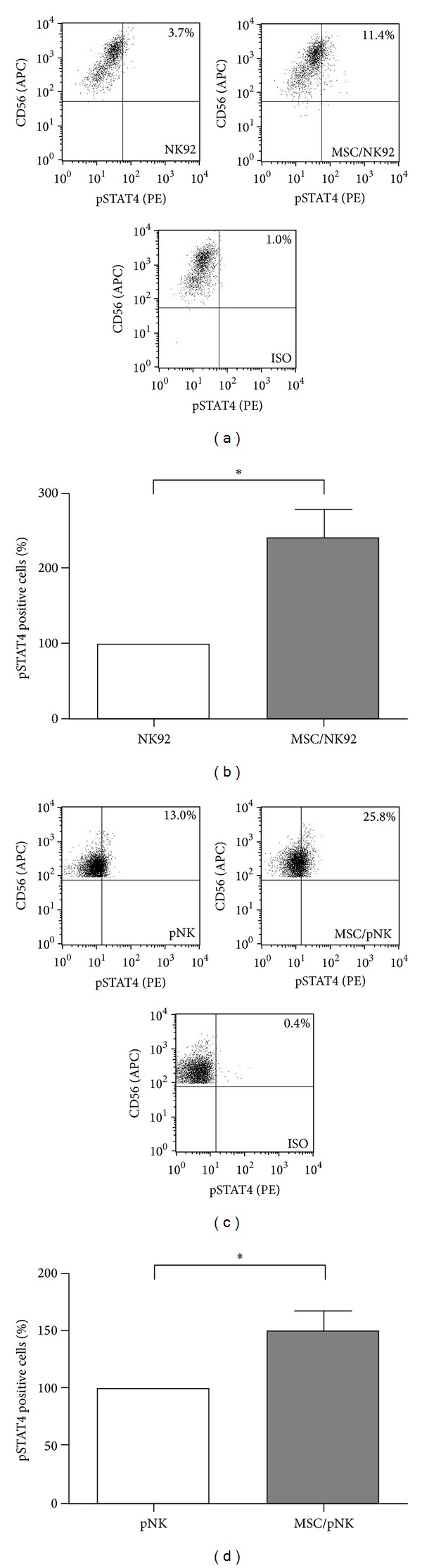
MSC stimulate the phosphorylation of STAT4 in NK cells. NK92 cells (a and b) and primary NK- (pNK) cells (c and d) were cultured in the absence or presence of MSC at a NK : MSC ratio of 5 : 1. Cells were preincubated for 24 h prior to stimulation with IL-12 (1 ng/mL) and IL-18 (10 ng/mL). Sixteen hours after stimulation, cells were stained with antibodies against CD56 and intracellular pSTAT4 (phosphorylated STAT4). Intracellular pSTAT4 levels were determined by flow cytometry. ((a) and (c)) Dot plots of one representative experiment. ((b) and (d)) Bar graph shows mean values ± standard deviation of 4 (b) and 6 (d) independent experiments performed using (b) 4 different donors for MSC and (d) 3 and 4 different donors for MSC and pNK cells, respectively. Data are normalized and presented as percentage of pSTAT4 positive NK cells cultured in the presence of MSC with respect to NK cells cultured alone (100%). *P < 0.05.

Moreover, intracellular phosphorylation of STAT4 was determined both in NK92 cells and in primary NK cells after stimulation with IL-12 and IL-18 either in the presence or in the absence of MSC. When cocultured with MSC 11.4% of NK92 cells expressed pSTAT4 in comparison to 3.7% of NK92 cells alone (Figure 5(a)). Phosphorylated STAT4 was detected in 25.8% and 13% of primary NK cells in the presence and absence of MSC, respectively (Figure 5(c)). Independently of the donor of MSC or primary NK cells this MSC-mediated phosphorylation of STAT4 was significantly enhanced in NK92 cells as well as in primary NK cells (Figures 5(b) and 5(d)). In summary, the stimulatory effect of MSC in terms of IL-12/IL-18-induced IFN-*γ* synthesis in NK cells was associated with a partially increased surface expression of the IL-12R*β*1 chain and an intracellularly enhanced phosphorylation of STAT4.

## 4. Discussion

In the present study, we show that MSC enhance the ability of NK cells to secrete IFN-*γ*. This modulatory activity of MSC was dependent on cell-cell contact as well as on MSC-derived soluble factors. Moreover, the MSC-mediated increase in IFN-*γ* secretion was associated with enhanced IL-12R expression and phosphorylation of STAT4.

Our data clearly demonstrate that IL-12/IL-18-stimulated NK cells were activated to secrete increasing amounts of IFN-*γ* in the presence of MSC (Figures 1(a) and 1(b), Table 1). This stimulating effect of MSC was observed not only with the human cell line NK92 but also with primary peripheral blood-derived NK cells (Figure 1(d), Table 1) and, therefore, might represent a relevant mechanism* in vivo*. Conflicting data exist about the mode of NK cell modulation via MSC, which to some extent seems to depend on the experimental conditions like the time interval for the coculture, the cell densities, or the nature of NK cell stimulation. The striking difference in all studies published yet was the kind of NK cell stimulation that was applied to the cultures in the absence or presence of MSC. Obviously, stimuli which are added before or during the coculture seem to have major influence on how NK cells respond to MSC. Interestingly, all studies described so far were done in the presence of IL-2, in part, in combination with other stimuli like FO-1 melanoma cells [25], T-cell depleted PBMC [21], or irradiated feeder cells together with phytohaemagglutinin [22]. To the best of our knowledge we show here for the first time that MSC potentiate the IFN-*γ* secretion by NK cells under special environmental conditions, namely, in the presence of the proinflammatory cytokines IL-12 and IL-18. Both cytokines are frequently expressed during the immune response to pathogens. Therefore, it is tempting to speculate that MSC when present at the site of infection, for example, in injured tissues, support the elimination of invading pathogens through priming of NK cells for enhanced IFN-*γ* production. In addition to the nature of NK cell stimulation the time interval for MSC and NK cell contact seems to have some minor influence on the NK cell modulation via MSC. A decreased IFN-*γ* production by NK cells was observed upon coculture with MSC for 24 h [20] as well as for extended cultures of 5 to 6 days [21, 25]. In contrast, enhanced NK cell derived IFN-*γ* production was described exclusively within the first 24 h of coculture with MSC [22–24]. We found a stimulating effect of MSC during the initial 24 h of coculture that persisted for at least 3 days (Figure 1(c)). Therefore, we exclude that the MSC-mediated activation of NK cell derived IFN-*γ* production was only an initial or a delayed event during the contact of MSC and NK cells.

Our transwell as well as transfer experiments with conditioned media from MSC show that, adjacent to cell-cell contact, soluble factors were also responsible for the stimulating effect of MSC on the IL-12/IL-18–induced IFN-*γ* secretion of NK cells (Figure 2). These so far unidentified factors seem to be expressed constitutively by MSC and in particular upon contact with NK cells, as we could observe a progressively enhanced IFN-*γ* secretion of NK cells upon stimulation with IL-12/IL-18 in conditioned media derived from MSC alone and from cocultures of MSC and NK cells (Figure 2(b)). Common soluble factors produced by MSC are, for example, IL-6, IL-8, IL-10, IL-12, prostaglandin E_2_ (PGE_2_), and transforming growth factor *β*1 (TGF-*β*1) [19]. Under specific conditions, for example, in response to viral infection, MSC produce also IL-2 [27]. Factors like IL-10, TGF-*β*, and PGE_2_ are known to counteract inflammation including the production of IFN-*γ* and, thus, most likely were not responsible for the stimulatory effect of MSC on NK cells that we show here. Potential candidates for such MSC-derived stimulatory factors might be IL-12 and/or IL-2 that represent known amplifier of NK cell-derived IFN-*γ* synthesis [28]. However, the nature of the stimulatory soluble factors in the conditioned media that are produced by MSC and possibly by NK cells as well remains unknown and is currently under investigation.

It is important to note that besides the secretion of soluble factors cell-cell contact between NK cells and MSC played a crucial role in the enhanced IL-12/IL-18-induced IFN-*γ* secretion by NK cells in the presence of MSC (Figure 2(a)). The most important activating receptors on the surface of NK cells are NKp30, DNAX accessory molecule-1 (DNAM-1), and NKG2D. The ligands for the receptors DNAM-1 and NKG2D and CD112/CD155 and MIC-A/ULBP-3, respectively, are expressed on MSC and interactions between these ligands and their receptors are known to be involved in the NK-mediated cytotoxicity against MSC [22]. We did not observe any lysis of MSC upon coculture with NK cells under our experimental settings. However, IFN-*γ* exposed MSC are protected against NK-mediated lysis probably due to upregulation of HLA class I molecules at the MSC surface [22]. Further studies are required to identify the receptor-ligand interactions that were responsible for the MSC-NK-cell contact-dependent increase of the IFN-*γ* secretion reported here.

Mechanistically, we identified alterations in the IL-12 signaling pathway including the expression of the IL-12 receptor as well as STAT4 phosphorylation in NK cells upon contact with MSC (Figures 4 and 5). The *β*1 chain of the IL-12 receptor was upregulated 2-fold on NK92 cells upon contact with MSC (Figures 4(a) and 4(b)). In contrast, on primary NK cells the IL-12 receptor *β*1 chain was increased only on NK cells of a subgroup of donors and therefore did not reach statistical significance (Figures 4(c) and 4(d)). We assume that a rising expression of IL-12R*β*1 on the surface on NK cells after coculture with MSC is not solely responsible for the observed enhanced IFN-*γ* secretion. No expression of the *β*2 chain on the surface on NK cells could be detected (data not shown). As both chains are necessary for a functional IL-12 receptor [6] and the NK cells used here were susceptible for IL-12/IL-18-mediated stimulation, it seems likely that the expression of *β*2 was too low for detection in our experimental setting but still significant for IL-12 signaling. Additionally, IL-12 and IL-18 activate the expression of the T-cell-specific T-box transcription factor (T-bet), which is involved not only in the production of IFN-*γ* [29, 30] but also in the transcription of its target gene* IL-12R*β*2* [31] that in turn enhances the sensitivity of NK cells to IL-12. STAT4 is the key transcription factor for IL-12-induced IFN-*γ* production [32]. Accordingly, we observed a more than 2-fold upregulation of pSTAT4 in NK92 as well as in primary NK cells (Figure 5). Therefore, we speculate that the presence of MSC allows in part an enhanced binding of IL-12 and an increased IL-12 signaling including increased phosphorylation of STAT4. Consequently, the percentage of NK cells that produced IFN-*γ* increased upon contact with MSC (Figure 3). Therefore, we suggest that MSC prime NK cells for increased IL-12 sensitivity by a still unknown mechanism resulting in a stimulation of NK cell mediated IFN-*γ* production and secretion.

Due to their regenerative properties MSC are therapeutically used in transplantation or tissue engineering approaches with the aim to improve the process of tissue regeneration. Moreover, due to their anti-inflammatory properties MSC are considered to have beneficial effects upon application in, for example, graft versus host disease, Crohn's disease, and multiple sclerosis [17, 18]. Additionally, it was shown recently that human MSC also exhibit antimicrobial effector functions that are induced by inflammatory cytokines [33]. Nevertheless, a key role in determining the success of using MSC in clinical trials may play the crosstalk between implanted donor MSC and recipient immune cells, and the outcome of this interaction is dependent particularly on the microenvironment of the cells involved. It is tempting to speculate that in T-cell-associated disorders like graft versus host disease, Crohn's disease, and multiple sclerosis MSC initially impair the proliferation and/or function of disease-promoting T-cells, whereas in tissue damage or infections MSC primarily interact with cells of the innate immune system like NK cells. As MSC and NK cells come in close contact within the injured tissue the presence of the proinflammatory cytokine IL-12 might trigger an enhanced level of IFN-*γ* that could influence immune defense mechanisms, tissue regeneration, and the function of MSC. As such, IFN-*γ* released by NK cells might inhibit bone repair mechanisms but otherwise might promote muscle healing [13–15, 34]. Moreover, increased levels of IFN-*γ* released from NK cells upon contact with MSC might support the immune response to invading pathogens at the site of tissue injury. However, there is increasing evidence that therapeutically applied MSC not only appear at the site of tissue injury but also mainly migrate to liver, lung, and spleen [35] with unknown consequences so far. The present study indicates that the appearance of therapeutically applied MSC in these NK-cell-rich organs might bear the risk of hyper inflammation in case of otherwise harmless infections.

## 5. Conclusions

MSC enhance the ability of NK cells to produce and secrete IFN-*γ*. This modulatory activity of MSC is mediated via cell-cell contact as well as by soluble factors and involves, at least in part, the STAT4 signaling pathway. As MSC and NK cells might come in close contact within injured tissues or in several organs after therapeutic application of MSC one should be aware that MSC obviously may not only limit inflammatory processes but also, according to our study, may even favor inflammation which may be detrimental for the host.

## Figures and Tables

**Table 1:** Summarized data of the MSC-mediated IFN-*γ* secretion of IL-12/IL-18–stimulated NK cells shown in Figure 1.

	IL-12/18 directly		IL-12/18 after 24 h	
	NK92/MSC (%)	pNK/MSC (%)	NK92/MSC (%)	pNK/MSC (%)
Figure 1(a) + Figure 1(b)				
20 : 1	182 ± 68**		211 ± 42**	
10 : 1	211 ± 91**		261 ± 66**	
5 : 1	249 ± 75**		459 ± 88**	
1 : 1	344 ± 37**		714 ± 94**	
Figure 1(c)				
24 h	343 ± 129**			
48 h	337 ± 102**			
72 h	448 ± 116**			
Figure 1(d)	334 ± 215*	216 ± 37**	543 ± 295**	485 ± 166**

Values are normalized to IFN-*γ*  secretion of stimulated NK cells only set as 100%.

Values are mean ± standard deviation of three independent experiments performed in triplicates and with three different donors of MSC.

**P < 0.01, *P < 0.05 significantly different from NK cells only set as 100%.
